# Extension and Limits of Depolarization-Fringe Contrast Roughness Method in Sub-Micron Domain

**DOI:** 10.3390/s21165572

**Published:** 2021-08-19

**Authors:** Franziska Pöller, Félix Salazar Bloise, Martin Jakobi, Jie Dong, Alexander W. Koch

**Affiliations:** 1 Institute for Measurement Systems and Sensor Technology, Technical University of Munich, Theresienstrasse 90, 80333 Munich, Germany; m.jakobi@tum.de (M.J.); jie.dong@tum.de (J.D.); a.w.koch@tum.de (A.W.K.); 2 ETSI Minas y Energía, Universidad Politécnica de Madrid, Calle de Ríos Rosas 21, 28003 Madrid, Spain; felixjose.salazar@upm.es

**Keywords:** roughness measurement, non-contact method, depolarization, roughness modeling, extension, limits

## Abstract

To guarantee quality standards for the industry, surface properties, particularly those of roughness, must be considered in many areas of application. Today, several methods are available on the market, but some damage the surface to be tested as they measure it by contact. A non-contact method for the precise estimation of sub-micron roughness values is presented, which can be used as an extension of existing roughness measurement techniques to improve them further considering the depolarized light reflected by the sample. This setup is based on a Michelson interferometer, and by introducing a quarter-wave plate on a half part of the reference mirror, the surface roughness can be directly derived by measuring the fringe contrasts. This article introduces a simple model describing the intensity distortions resulting from the microscopic roughness in divided interferograms when considering depolarization. This work aimed to extend the measurement range of the technique developed in a previous work, in which depolarization effects are taken into account. For verification, the experimental results were compared with the fringe contrast technique, which does not consider the depolarization of the scattered light, especially regarding the extended wavelength interval, highlighting the limits of the technique. In addition, simulations of the experiments are presented. For comparison, the reference values of the sample roughness were also generated by measurements with a stylus profiler.

## 1. Introduction

Due to the increasing requirements of the industry, it is important to accurately characterize a surface. This requires precise knowledge of the roughness of the surface. Surface roughness plays a role in many different applications: in the machining of workpieces [1] such as honing, the surface roughness has an impact on the quality [2,3]; in pharmacy, the surface roughness of the film coating of tablets influences drug release [4,5]; components in the automotive industry and for aerospace applications must have a specific surface roughness [6,7]; in medicine, surface roughness affects the biocompatibility of an implant [8,9].

There are mainly two ways to determine the roughness of a surface, namely contact and non-contact. As the demands on a surface increase in industrial processes, it must also be ensured that the sample is perturbed as little as possible during a roughness measurement. Since reliable contact methods, such as the mechanical profilometer, can damage the surface with their stylus tips, such as rubbers and plastics, this is also a time-consuming process to perform. This motivates research on alternative measurement techniques for roughness determination that are faster, accurate, and at the same time, applicable in more extensive kinds of samples.

In addition, the various methods for determining the surface roughness can be divided into three categories: the so-called profiling techniques, topographical methods, and area-integrated procedures [10].

The stylus profilometer, which belongs to the profile techniques, requires a profile of the surface $z\left(x\right)$ to evaluate the roughness. This method provides reliable results, but measuring a surface takes a significance amount of time because the sample has to be measured and moved line by line. In addition, the width of the stylus tip does not allow the resolution of arbitrarily small structures [11,12,13]. On the other hand, topographical methods produce a result that is made up of several parallel lines and produce result images $z\left(x,y\right)$. This type of measurement includes coherence interferometry, such as white light interferometry [14], scanning probe microscopy (e.g., using an atomic force microscope (AFM) [15] or a laser confocal microscope [16]), fringe projection [17], and focus variation microscopy [18]. Due to their scanning properties, a surface roughness distribution is obtained, but they are associated with high acquisition and maintenance costs. The surface integrating methods, represented by the speckle correlation [19] and the light scattering method [20], consider the surface microstructure and provide a statistical value for the roughness.

In a previous paper [21], we demonstrated an improvement of the roughness measurement up to approximately 40 nm, by considering the depolarization of the scattered light from a rough surface when employing the fringe contrast method [22]. In this context, this article aimed to extend the applicability of the technique to samples with higher roughness but in the submicrometer range by changing the wavelength of the radiation used. In this aim, in order to clarify the validity range and limitations of the method, measurements and simulations with the presented model were performed with different wavelengths showing the enlargement of the roughness measurement interval. The measurements were performed by inserting a quarter-wave plate (QWP) into a part of the reference beam of a Michelson interferometer. The resulting different contrasts, which depend on the depolarization of the laser light and the roughness of the surface to be inspected, can then be evaluated and the roughness estimated.

This technique can be used as an extension of roughness measurement methods where roughnesses in sub-micron ranges are required to guarantee high-quality surfaces, for which there is an increasing demand in industrial applications. The monitoring and precise knowledge of surface roughness are essential, e.g., for coatings. To name only a few examples, the coatings of solar cells must show a roughness in the sub-micron range [23]; the determination of the roughness of coatings, e.g., of smart glass can also be carried out by this method [24,25]; which also works for determining the thin-film coating hardness of components destined for the aerospace industry [26]. In addition, the presented method can be used for biomaterials in the medical sector [27,28].

The structure of this article is as follows. After a brief description of the theoretical approach of the depolarization-based roughness measurement (DBRM) method, on which the model is based, the optical setup, the Stokes parameters and the simulation model were explained. After that, in Section 3, to validate the method, the results of the experiments and of the simulations are shown. To conclude this paper, a discussion of the technique and its limitations are presented.

## 2. Theoretical Approach and Simulations

One of the effects resulting from the interaction between a coherent beam and a random medium is the change of the polarization state of the scattered light [29]. This fact can be a drawback in different measurement optical techniques where the change of the polarization of the scattered beam affects the formulae used describing a process, i.e., when the vectorial nature of the light cannot be neglected. An example of these techniques is that of the roughness measurement using the fringe contrast obtained from an interferogram [22]. In this case, disregarding the depolarization effects leads to obtaining larger roughness values than the actual ones. Thus, the consideration of polarization in approaching the problem becomes important, sometimes even essential, to obtain a limited (or bounded) value of roughness. Bearing this fact in mind, in the context of roughness, an interferometric procedure to improve roughness measurements in intervals of tens of nanometers is proposed [21]. In order to succinctly explain the basis of the method, we can describe it as follows.

### 2.1. Theory

When a linearly polarized electromagnetic wave interacts with a random medium, a complex speckle field is produced. Considering the fact that the polarization of the scattered field changes in every point P(x,y,z) of the space referring to a coordinate frame, the speckle may be understood as the incoherent superposition of two intensities, each one corresponding to two perpendicular electric components. In other words, this random intensity is similar to the sum of two independent linearly polarized speckle patterns, because two linearly polarized electric fields in mutually perpendicular directions do not produce any interference.

**Figure 1:**
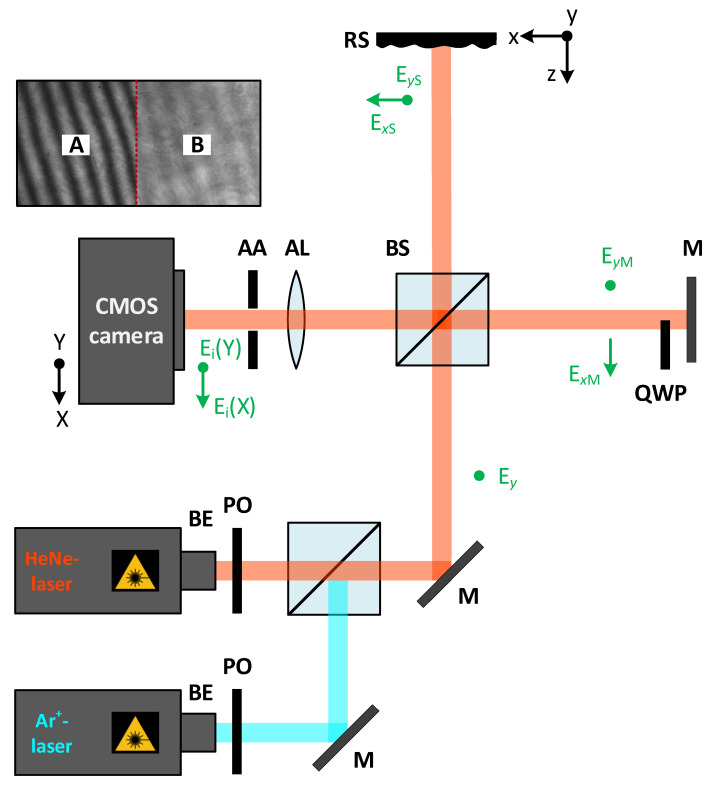
Schematic structure of the experimental setup with the electric field components at different positions (in green) and the resulting interferogram. BE: beam expander; PO: polarizer; M: plane mirror; BS: (non-polarizing) beam splitter; RS: rough surface; QWP: quarter-wave plate; CMOS: complementary metal-oxide semiconductor; AA: adjustable aperture; AL: achromatic lens.

Starting with this picture, let us suppose a laser beam linearly polarized in the direction of the *y* axis (see Figure 1). The electric field ${\vec{E}}_{0}=\left(0,E_{y0}\right)$ hits the rough surface (RS) after going through the non-polarizing beam splitter (BS), resulting in two non-zero components for the scattered electric field, i.e., ${\vec{E}}_{s}=\left(E_{xs},E_{ys}\right)$. At the same time, the beam impinges the mirror *M* of the interferometer on which a quarter-wave plate (QWP) has been placed only at a one-half part of it. Locating a quarter-wave plate enables one to obtain two perpendicular separately electric fields after beam reflection, namely ${\vec{E}}_{M}^{A}=\left(0,E_{yM}\right)$ (part A), and ${\vec{E}}_{M}^{B}=\left(E_{xM},0\right)$ (part B)—one of them corresponding to a part of the mirror. Thus, the resulting field amplitude on the detector is separated into two parts of the same picture. Considering the polarization variation of the reflected light from the sample, different contrasts $C_{A}$ and $C_{B}$ of the respective interference patterns on both sides A and B will be obtained. By measuring these contrasts of the interference fringes of both parts, the roughness of the sample may be more accurately calculated using the following formula [21]:

(1) $$\sigma =\frac{\lambda }{4\pi }\sqrt{\ln\left(\frac{4I_{M}I_{S}}{{\left(I_{xs}+I_{ys}+I_{M}\right)}^{2}\left(C_{A}^{2}+C_{B}^{2}\right)}\right)},$$

where $I_{S}=I_{xs}+I_{ys}$ represents the sum of the intensities *x* and *y*, $I_{M}$ is the intensity from the mirror, and λ is the wavelength of the radiation used. This formula corresponds to the root mean square (RMS) roughness $S_{q}$ [30]:

(2) $$Sq=\sigma_{z}.$$

### 2.2. Optical Setup and Measurement Procedure

For the simulations and measurements, the modified Michelson interferometer shown in Figure 1 is used, in which a quarter-wave plate (QWP) is partially placed in the reference beam. This design is also the basis for modeling the depolarization-induced intensity distortions of the reflecting sample, where the assumed parameters are identical to the values of the components in the experimental setup.

In order to ensure the initial polarization conditions, the collimated and expanded laser beam goes through a polarizer (PO). The used light source is a red HeNe-laser head (THORLABS) with the main laser line $\lambda_{\mathrm{HeNe}}=632.8\;nm$ and an output power of 12 mW, in addition to an Ar^+^-laser head (LEXEL 3500) with a laser line of the wavelength $\lambda_{\mathrm{Ar}+}=488.0\;nm$ and a wavelength-dependent single-line power of 1100 mW, respectively. The laser is applied in a single longitudinal operation. The Ar^+^-laser head is operated via an etalon (model LEXEL 3503). The 50:50 non-polarizing beam splitter (BS) splits the beam into an object path and a reference path. The laser beam, linearly polarized in the *y* direction, is directed onto the tilted plane mirror (M) in the reference path, where a quarter-wave plate is placed in one of the two halves, which we call part B (the other half without QWP is called part A). The plane mirror is slightly tilted in order to obtain interference fringes on the camera. The optical axis of the QWP is set to $45{}^{\circ }$ to generate, firstly, the circular polarized light [31,32]. This allows having the two necessary and different polarization states of the reference beam, which provide distinct information on part A and part B by the CMOS camera (Photonfocus) with a 1312×1082 pixels resolution and an 8 μm×8 μm pixel size corresponding to a resolution of 12 bit. It would be the same as having two Michelson interferometers integrated into one system. This setup was simplified by the QWP, which makes it, e.g., more space-saving. Simultaneously, the rough surface (RS) in the object path scatters the laser light, generating various interferometric fringe patterns on the two halves A and B of the camera. The achromatic lens (AL) with a focal length of f=80 mm and the adjustable aperture (AA) produce a sharp image of the object and reference the camera. The magnification of the measuring system is M=1.5.

To accurately determine the roughness of the sample, it is necessary to generate a set of interferometric patterns according to the theory described in Section 2.1. In order to obtain a value for the RMS roughness $S_{q}$, which is calculated in Equation (1), four steps must be performed, which also apply to the simulations: (i) in the first step, the total intensity of the rough surface $I_{S}$ is determined. For this, only the object path in Figure 1 is considered. The light scattered back from the surface (RS) is recorded with the CMOS camera, from which the intensity reflected from the rough surface, $I_{S}=I_{xs}+I_{ys}$, is evaluated; (ii) secondly, the total intensity of the reference mirror (M) $I_{M}$ is identified. The same intensities from both parts of the plane mirror are assumed to be, approximately $I_{M}=I_{xm}\approx I_{ym}$. For this measurement, only the reference path in the Michelson interferometer is regarded and the intensity reflected from the mirror (M) $I_{M}$, captured in another pattern, is recorded and assessed. To ensure the same intensity of the reference mirror on both parts, we place a glass in front of the mirror (in part A) to compensate for the beam intensity loss of the QWP (part B) due to reflections on the boundary surfaces (in the forward and back direction). Thereby, the (non-birefringent) glass must have similar reflection properties as the QWP. (iii) In the third step, both paths of the Michelson setup are considered, producing two interference patterns with different contrasts $C_{A}$ and $C_{B}$, resulting from the QWP, generated on the camera in a fringe pattern. These two contrasts in the fringe pattern are calculated according to the Michelson contrast for parts A and B, respectively. (iv) If the obtained values for $I_{S}$, $I_{xs}$, $I_{ys}$, $I_{M}$, $C_{A}$, and $C_{B}$ are inserted into Equation (1), the RMS roughness Sq of the rough surface yields. The recording of the series of the interferometric patterns (according to step (i) to (iii)) only requires approximately 5–10 s in total.

### 2.3. Degree of Polarization

As mentioned earlier, the interaction between a rough sample and the coherent radiation results in a depolarization of the scattered light. In our case, the existence of a fringe contrast in part B is a manifestation of this fact. To quantify this assumption, the degree of polarization (DOP) for each sample in the simulations, as well as in the experiments, is determined. Thus, the part of the electromagnetic wave which is polarized is known through a measure for the depolarization due to the rough surface. To some extent, we can relate the DOP with the roughness of the surface. The increase in the roughness improves the probability of multiple scattering effects, leading to greater fluctuations of the electric field components, and then changing the polarization of the reflected beam. So, we can intuitively say that a higher surface roughness means higher depolarization effects.

**Figure 2:**
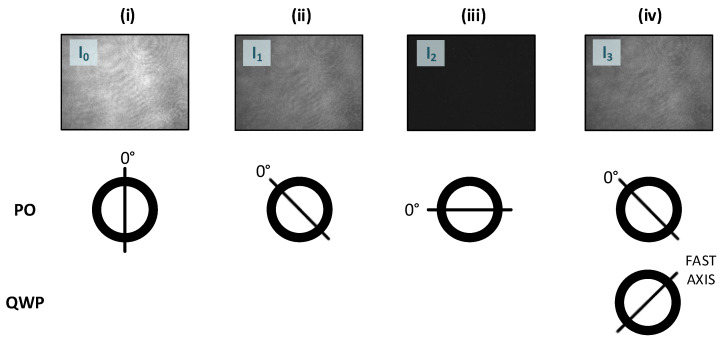
The generation of the Stokes parameters $S_{0}$, $S_{1}$, $S_{2}$, and $S_{3}$ to determine the degree of polarization (DOP) (with the adjustments of the polarizer (PO) and the quarter-wave plate (QWP) for each measurement): (**i**) capture of the intensity $I_{0}$ (first measurement); (**ii**) capture of the intensity $I_{1}$ (second measurement); (**iii**) capture of the intensity $I_{2}$ (third measurement); (**iv**) capture of the intensity $I_{3}$ (fourth measurement) (measurement pictures of a surface with the RMS roughness $S_{q}=31\;nm$ (see Sample 2 in Table 1) at a wavelength of λ=632.8 nm).

For calculating the degree of polarization, the Stokes parameters are determined first. Since the Stokes parameters can establish a relationship between the polarization of light and the intensity of light, these parameters can be used to describe the polarization state of light. The Stokes parameters can be easily measured without modifying the interferometric setup by only properly using a polarizer (PO), a quarter-wave plate (QWP), and the object path (cf. Figure 2). This gives the four different intensities required $I_{0}\left(0{}^{\circ }\right)$, $I_{1}\left(45{}^{\circ }\right)$, $I_{2}\left(90{}^{\circ }\right)$, and $I_{3}\left(45{}^{\circ }+\mathrm{QWP}\right)$. The Stokes parameters denoted by $S_{0}$, $S_{1}$, $S_{2}$, and $S_{3}$, can be expressed as a function of the measured intensities [33]:

(3) $$\begin{array}{cc} S_{0} & =I_{0}+I_{2} \end{array}$$

(4) $$\begin{array}{cc} S_{1} & =I_{0}-I_{2} \end{array}$$

(5) $$\begin{array}{cc} S_{2} & =2\;\cdot \;I_{1}-I_{0}-I_{2} \end{array}$$

(6) $$\begin{array}{cc} S_{3} & =2\;\cdot \;I_{3}-I_{0}-I_{2}. \end{array}$$

and the degree of polarization is calculated by:

(7) $$DOP=\frac{\sqrt{S_{1}^{2}+S_{2}^{2}+S_{3}^{2}}}{S_{0}}\leq 1.$$

### 2.4. Simulations

In order to prove the validity of the proposed procedure in order to extend the measurement interval of the technique, a set of computer simulations for the two wavelengths used in the experiments in addition to laboratory experiments were performed. In this aim, bearing in mind a usual image system based on coherent illumination, the actual electric field at the image plane $E_{i}\left(X,Y\right)$ corresponding to an object surface may be expressed as the convolution of the ideal image field amplitude $E\left(X,Y\right)$ and the impulse response for the optical system $h\left(X,Y\right)$:

(8) $$E_{i}\left(X,Y\right)=h\left(X,Y\right)*E\left(X,Y\right),$$

where the bracket $\left(X,Y\right)$ denotes the coordinates on the image plane, and the ∗ is the convolution operator. In Equation (8), $E\left(X,Y\right)$ contains the information of the field distribution on the rough surface, and $h\left(X,Y\right)$ is the Fourier transform of the lens aperture of diameter *D*, where:

(9) $$h\left(X,Y\right)=\frac{D}{2}\frac{J_{1}\left(\pi Dq\right)}{q},$$

with $q=\frac{\rho }{\lambda z}$. The function $J_{1}$ represents the first-order Bessel function of the first kind, λ the wavelength of the laser, ρ the distance between the origin of the coordinate frame XY and an arbitrary point on this system, and *z* the distance between the lens and the image plane. Obtaining $E\left(X,Y\right)$ throughout Equation (9) is usually a hard task; however, it is easier to perform this calculation using the Fourier transform. In effect, the relationship in Equation (8) is equivalent to the following formula:

(10) $$E_{i}\left(X,Y\right)=FT^{-1}\left\{FT\left(h\right)\;\cdot \;FT\left(E\right)\right\},$$

where *FT* stands for the Fourier transform, and $FT^{-1}$ for its inverse.

The rough sample to be investigated is modeled like a random two-dimensional reflectance mask composed of N×N elements, each of which represents the decomposition of the surface heights into its single elements. The model assumes a coherent illumination and plane waves, with the diffusing surface (DS) being Gaussian distributed.

Let us suppose that the sample is illuminated with linearly polarized light. The distribution of heights produces random variations in the phases of the diffracted field, leading to well-known speckles. As mentioned before, the interaction between the impinging electric field and the sample leads to changes in the polarization state of the reflected waves. To obtain a mathematical expression of these waves immediately behind the surface under test, each component of the field scattered by the surface $E_{\mathrm{DS}}^{s}$ (s=x,y) can be expressed by a complex exponential phase term which includes the behavior of this field at every point (discretized for simulation) on the rough surface:

(11) $$\begin{array}{cc} E_{\mathrm{DS}}^{s}\left(x_{k},y_{l}\right) & =E_{0}^{s}\left(x_{k},y_{l}\right)\;\cdot \;\exp\left(2\pi i\left(S\left(x_{k},y_{l}\right)+R\left(x_{k},y_{l}\right)\right)\right), \end{array}$$

(12) $$\begin{array}{cc} s & =x,y, \end{array}$$

where $E_{0}^{s}\left(x_{k},y_{l}\right)$ is the amplitude very close to the point $\left(x_{k},y_{l}\right)$, $S(x_{k},y_{l})$ is a function representing the whole smooth surface (very low spatial frequencies), and $R(x_{k},y_{l})$ is the random distribution taken for the roughness (high spatial frequencies). Both functions *S* and *R* must be fitted in the program (code) accordingly for each roughness measured. Observe that Equation (11) considers media that can generate random amplitudes, which should be included in the term $E_{0}^{s}\left(x_{k},y_{l}\right)$. This could be the case, for instance, of a surface where its reflecting properties vary from point to point. In the studied case, without loss of generality, we do not take different values for $E_{0}^{s}$ on the entire surface.

For the reference path, a reflection mask must also be defined for the plane wave reflected by the reference mirror (M) on both parts A and B. This wave may be expressed as

(13) $$\begin{array}{cc} E_{M}^{p} & =E_{0}^{p}\;\cdot \;\exp\left(2\pi i\mathrm{OP}\right), \end{array}$$

(14) $$\begin{array}{cc} p & =A,B, \end{array}$$

where OP represents the optical path, and $E_{0}^{p}$ is the amplitude.

For the simulations, the three measurements (i), (ii), and (iii), as described in Section 2.2, have to be conducted. Thus, the recordings of the CMOS camera for further processing is implemented, where the theoretical approach of the DBRM method is applied. For this, the intensities, namely the total intensity of the rough sample $I_{S}$, the total intensity of the reference mirror $I_{M}$, and the two different contrasts on the interferometric fringe pattern $C_{A}$ and $C_{B}$ must be generated.

**Figure 3:**
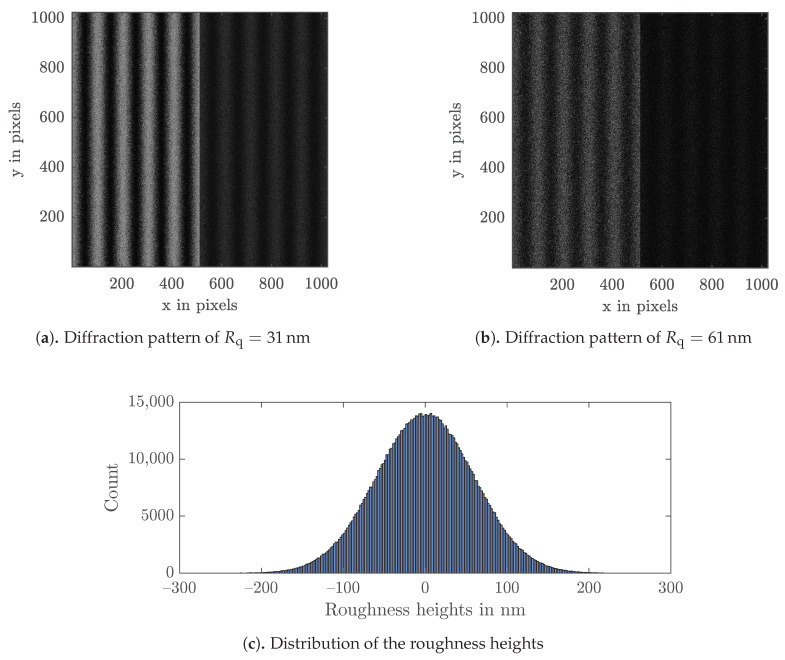
2D model for the wavelength λ=632.8 nm.

First, by considering the two components of the diffracted field from the sample $\left(E_{\mathrm{DS}}^{x},E_{\mathrm{DS}}^{y}\right)$, the total intensity $I_{S}$ is simulated. Considering that these two electric fields are uncorrelated, the total intensity on the detector for this step of the procedure is the addition of the intensities associated with both components similarly to independent phenomena, that is $I_{S}=|E_{\mathrm{DS}}^{x}\left(x_{k},y_{l}\right){|}^{2}+{\left|E_{\mathrm{DS}}^{y}\left(x_{k},y_{l}\right)\right|}^{2}$. As a second step, the total intensity of the reference $I_{M}$ is modeled by simply regarding the plane wave from the mirror $I_{M}={\left|E_{M}^{s}\left(x_{k},y_{l}\right)\right|}^{2}$. For the interferometric fringe pattern simulation with the two different contrasts $C_{A}$ and $C_{B}$, both paths of the interferometer setup are considered. Thus, the two fields $E_{\mathrm{DS}}^{x}$ and $E_{M}^{x}$ on part A, and $E_{\mathrm{DS}}^{y}$ and $E_{M}^{y}$ on part B, interfere independently, resulting in two speckle fringe patterns. This is also clearly visible in the simulated interferograms for two exemplary roughnesses of $R_{q}=31\;nm$ and $R_{q}=61\;nm$, with the two contrasts $C_{A}$ (left in Figure 3a,b) and $C_{B}$ (right in Figure 3a,b), where a wavelength of λ=632.8 nm was assumed. Figure 3c shows the corresponding Gaussian distribution of roughness deviations from the mean height.

For the simulations, the same lens parameters in the Michelson interferometer in Figure 1 were assumed as for the experiments, namely the diameter of the achromatic lens D=20 mm and the focal length f=80 mm. The sample under test was squared with a side length of L=2 cm, which corresponds to a pixel number of 1024×1024, defining the sample size [34]. Therefore, the rough surface is represented by a 2D sampled grid of pixels.

Additionally, to quantitatively assess the depolarization effects of the simulated roughnesses, a simulation of the degree of polarization was performed and evaluated using Equations (3)–(6). The four different positions of the polarizer or QWP in front of the imaging optics were performed in different positions to account for the values of the intensities needed. These intensities enable one to determine $S_{0}$, $S_{1}$, $S_{2}$, and $S_{3}$ and then the degree of polarization (DOP) for each simulated surface.

## 3. Results

To verify the extended applicability of the method to higher roughness values with further wavelengths, seven reflective flat samples with different Gaussian distributed roughnesses were employed ($S\left(x,y\right)=0$). The laser wavelengths 488.0 nm of an Ar^+^ laser head and 632.8 nm of a red HeNe laser head were chosen to obtain a broad spectrum within which the method can be applied. In order to show the improvements of the DBRM technique and its limits, results without considering depolarization effects (Chandley’s method [22]) and with the new procedure are presented and compared. Together with all the realizations, simulations of the experiments are made. In addition, to be sure that the method provides valid roughness results in the order of magnitude, a commercial stylus instrument was used to measure the roughness of the specimens.

### 3.1. Experimental Results

To verify the increase in the measurement range by this method (DBRM), several experiments were performed using the setup shown in Figure 1.

Measurements of the first four samples and the seventh sample with a wavelength of $\lambda_{1}=488.0\;nm$ have already been performed in [21], and it has been shown that small roughnesses can be correctly and more accurately determined, with respect to Chandley’s technique. To see the improvements by changing the wavelength and to discuss them, the experimental results of [21] are again included in Table 1.

To demonstrate the increment in the measurement interval, we performed further experiments with a second wavelength, namely $\lambda_{2}=632.8\;nm$, and with two additional samples (samples 5 and 6 (see Table 1)). Since the experimental results using the Michelson interferometer (according to Equation (1)) may vary slightly from one part of the surface to another, the $Sq_{\mathrm{DBRM}}$ values and the $Sq_{\mathrm{WODSL}}$ values from nine different areas for each sample were calculated and averaged.

To determine the order of magnitude of the roughness, we conducted additional reference measurements with a stylus profiler as a possible reference. We measured different profile lines of the samples twenty times in different directions using a stylus instrument (SURFCOM FLEX 50 A with a measuring force of 0.75 mN, a stylus tip radius of 2 μm, and an uncertainty of 1.6 nm) and then averaged them. By this average, the one-dimensional parameter for the RMS roughness $R_{q}$ of the stylus profiler can be equated with the two-dimensional RMS roughness $S_{q}$ resulting from the DBRM method in Equation (1). All these results are depicted in Table 1.

These values show two important findings. Firstly, the differences between the roughness values provided by Chandley’s method [22] and the new technique are noted. The results considering depolarization effects are more similar to those given with the stylus; and secondly, with the wavelength of $\lambda_{2}=632.8\;nm$, the roughness measurements can be extended up to 61 nm (Sample 6: DBRM-$\lambda_{2}$ in Table 1). Thus, using $\lambda_{2}=632.8\;nm$, the roughness measurement range (relative to wavelength $\lambda_{1}=488.0\;nm$) shifts the left and right limits, extending the measuring range to higher roughnesses. The method up to the seventh specimen fails, where the differences between the results by the DBRM and the first row (stylus) are noticeable.

**Figure 4:**
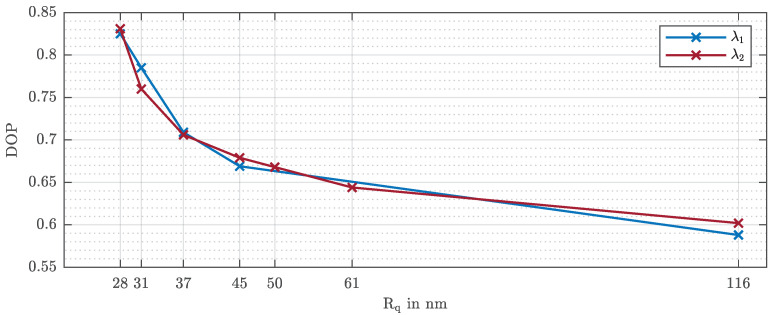
Measured degrees of polarization (DOPs) for $\lambda_{1}=488.0\;nm$ [21] and $\lambda_{2}=632.8\;nm$.

In order to measure the depolarization and to be able to quantify the portion of light polarized by the rough sample, the degree of polarization for each of the seven samples was also determined. In order to avoid some problems derived from the surface anisotropy, different areas were measured and the results averaged. The measured degrees of polarization (DOPs), evaluated according to Equation (7), for the wavelength $\lambda_{1}=488.0\;nm$ [21] and the wavelength $\lambda_{2}=632.8\;nm$ are graphically shown in Figure 4. As expected, the DOP values for both wavelengths decrease with increasing roughness since a higher microscopic roughness depolarizes more light. Since the Stokes’ parameters $S_{0}$, $S_{1}$, $S_{2}$, and $S_{3}$ differ slightly for different wavelengths and the DOP is theoretically defined as the quotient of the Stokes’ parameters, the curves in Figure 4 have small deviation with respect to each other.

To determine the uncertainties of the measurements in Table 1, the expanded uncertainty was calculated as in [21]. The uncertainties of the stylus profiler in Table 1 are given by the instrument itself and are specified according to the manufacturer’s information. From Table 1, there is an uncertainty of 4 nm from the seventh sample at a wavelength of 632.8 nm (Sample 7: DBRM-$\lambda_{2}$ in Table 1), a small uncertainty of 2 nm starting at the third sample (Sample 3: DBRM-$\lambda_{1}$ in Table 1), and a larger uncertainty of 3 nm (Sample 4: DBRM-$\lambda_{1}$ in Table 1) and 4 nm (Sample 7: DBRM-$\lambda_{1}$ in Table 2), respectively, at a wavelength of 488.0 nm.

These results reflect the limit of roughness up to which the DBRM method correctly estimates the roughness of the surface and thus has its validity in the visible range. In effect, considering the wavelength of 488.0 nm as light in proximity of the lowest wavelength we could have in the laboratory (Ar^+^-laser) and the highest corresponding to a red laser (HeNe), the limits of the technique can be stabilized.

At a wavelength of 488.0 nm, small roughnesses in the range of Rq=28 nm (Sample 1 in Table 1) to Rq=37 nm (Sample 3 in Table 1) can be estimated with high accuracy, at the laser wavelength 632.8 nm; this limit is extended, and slightly larger roughnesses up to Rq=61 nm (Sample 7: DBRM-$\lambda_{2}$ in Table 1) can be correctly evaluated.

Therefore, our method is not limited to a single wavelength and can also be applied to another wavelength in practice. The results of the experiments and the simulations deviate slightly to the right (towards the rougher samples) as the wavelength decreases. Taking into account all the results above, we can conjecture that the upper measurement limit of the DBRM method for any wavelength in the visible interval is $\left(\frac{\lambda }{R_{q}}\right)\approx 0.01$.

### 3.2. Simulation Results

For a more accurate statement about the behavior of sub-micron roughnesses taking depolarization into account, simulations were performed, the results of which were supported experimentally. For this purpose, seven reflecting surfaces whose roughness values correspond to the experimentally studied samples were generated. The simulations were performed and evaluated with the same wavelengths used in the experiments and using the same experimental setup as in the laboratory. The wavelengths each chosen within the visible wavelength range with the lower limit of $\lambda_{1}=488.0\;nm$ and the upper limit of $\lambda_{2}=632.8\;nm$, being produced with real laser sources in the experiments. The behavior of the rough samples considering their respective depolarizations at the two cutoff wavelengths of $\lambda_{1}=488.0\;nm$ and $\lambda_{2}=632.8\;nm$ were additionally confirmed experimentally.

Comparing the simulations (in Table 2) with the experimental results presented in the last section, we see that they agree. So, by considering depolarization in the physical process, and using a higher wavelength ($\lambda_{2}=632.8\;nm$), an improvement of the roughness values compared with Chandley [22] is obtained. In addition, with a larger wavelength, higher roughness values can be estimated, as highlighted by the experiments. Again, up to the seventh sample, the method fails, then the limit at which the DBRM method works can be established.

The simulated intensities and roughnesses of the sample, according to the model, try to be as close as possible to the reality of the physical properties. However, small deviations between the measurements and the simulations can occur due to some factors, such as deviations of the surface height distribution, or small changes in the reflectivity of the sample.

As mentioned above, the degree of polarization (DOP) is a direct indicator of the roughness of the rough surface.

For a better understanding of the experimental results and to confirm the measurements of the degree of polarization (see Figure 4), the DOP of the scattered light by the seven different samples generated according to the model, for the wavelengths $\lambda_{1}=488.0\;nm$ and $\lambda_{2}=632.8\;nm$, were simulated (see Table 2).

## 4. Discussion

In summary, the DBRM method is not limited to a certain wavelength but can be used in the entire visible spectrum to supplement existing roughness measurement techniques. The measurements have allowed us to measure the limits of the visible wavelength range and show that the small roughness of the samples up to 61 nm can be correctly determined with higher accuracy, and the roughness range, in which the method can be applied, is shifted with a higher wavelength of 632.8 nm. With this wavelength, the roughness of two rougher samples can be correctly measured compared to a shorter wavelength of 488.0 nm (see Table 1). By additional simulations for the wavelengths used in the experiments, this shift of the boundary is made even clearer (see Table 2). In general, the simulation results and the measurement results agree well, and the expected trend can be seen, which allowed us to verify the DBRM method in this article. With a short measurement time of a few seconds and increased accuracy by taking into account the depolarization of the sample, the proposed method is suitable for all reflective materials. The DBRM technique represents a cost-effective measurement method for optical surface characterization in the industry and can be applied as a supplementary method of roughness measurement in optical systems. Therefore, the technique is attractive for improving existing methods in the field of nano-finishing or surface coatings for biomaterials in the medic sector or for photovoltaic systems, which are indispensable for the energy revolution. The measurement setup is simple, and the measurement is easy to perform. However, it must be ensured that the QWP is set very accurately (tolerance angle of $2{}^{\circ }$); otherwise, erroneous results will be produced. The optimization of the optical components can further improve the results. In addition, the limits of the method can be defined more precisely by further experiments, and the application area can be specified even more precisely. In the future, it would be conceivable to not only measure metallic materials as demonstrated here, but other materials such as coatings for IR optics would also be feasible to become even more application-specific, where the experiments have to be carried out with infrared wavelengths and special optics for the infrared range are required in the interferometric setup, such as mirrors, lenses, or the camera.

## Figures and Tables

**Table 1:** Experimental results for seven samples measured by the stylus profiler, Chandley’s method without considering the depolarization of the scattered light (WODSL), and the DBRM method with $\lambda_{1}=488.0\;nm$ and with $\lambda_{2}=632.8\;nm$ considering the depolarization of the samples. Additionally, the uncertainties of the measurements are provided.

	Sample 1	Sample 2	Sample 3	Sample 4	Sample 5	Sample 6	Sample 7
stylus profiler ($R_{q}$)	28±2nm	31±2nm	37±2nm	45±2nm	50±2nm	61±2nm	116±4nm
WODSL—$\lambda_{1}$ ($S_{q}$) [21]	40±2nm	41±2nm	44±2nm	44±2nm	—	—	49±1nm
DBRM—$\lambda_{1}$ ($S_{q}$) [21]	27±1nm	31±1nm	32±2nm	28±3nm	—	—	31±4nm
WODSL—$\lambda_{2}$ ($S_{q}$)	46±3nm	46±3nm	48±2nm	55±2nm	64±2nm	69±2nm	55±2nm
DBRM—$\lambda_{2}$ ($S_{q}$)	27±1nm	31±1nm	36±1nm	44±1nm	50±1nm	59±1nm	48±4nm

**Table 2:** Simulation results for seven (reflecting) samples according to Chandley’s method without considering the depolarization of the scattered light (WODSL) [22] and according to the model describing the DBRM technique with $\lambda_{1}=488.0\;nm$ and $\lambda_{2}=632.8\;nm$. Moreover, the uncertainties of the measurements (see Table 1) and the simulated degrees of polarization (DOPs) for $\lambda_{1}$ and $\lambda_{2}$ are shown.

	Sample 1	Sample 2	Sample 3	Sample 4	Sample 5	Sample 6	Sample 7
stylus profiler ($R_{q}$)	28±2nm	31±2nm	37±2nm	45±2nm	50±2nm	61±2nm	116±4nm
WODSL—$\lambda_{1}$ ($S_{q}$)	32nm	32nm	42nm	43nm	47nm	56nm	39nm
DBRM—$\lambda_{1}$ ($S_{q}$)	28nm	31nm	35nm	37nm	40nm	30nm	22nm
WODSL—$\lambda_{2}$ ($S_{q}$)	33nm	33nm	40nm	45nm	50nm	59nm	39nm
DBRM—$\lambda_{2}$ ($S_{q}$)	28nm	31nm	37nm	44nm	49nm	55nm	24nm
DOP—$\lambda_{1}$	0.833	0.789	0.711	0.674	0.645	0.624	0.598
DOP—$\lambda_{2}$	0.839	0.743	0.703	0.668	0.657	0.639	0.610

## Data Availability

Data is contained within the article.
